# Assessment of Temporomandibular Disorders, Oral Health Status, Knowledge and Hygiene Behaviours Among Athletes in Croatia: A Cross-Sectional Study

**DOI:** 10.3390/epidemiologia7010006

**Published:** 2026-01-04

**Authors:** Josip Kapetanovic, Ivan Lucin, Ivan Kovacic, Antonija Tadin

**Affiliations:** 1 Department of Oral Surgery, School of Medicine, University of Mostar, 88000 Mostar, Bosnia and Herzegovina; josip.kapetanovic@mef.sum.ba; 2 Department of Endodontics and Restorative Dental Medicine, Study of Dental Medicine, School of Medicine, University of Split, 21000 Split, Croatia; ivan.lucin15@gmail.com; 3 Department of Dental Medicine, Department of Maxillofacial Surgery, University Hospital of Split, Spinciceva 1, 21000 Split, Croatia; 4 Department of Prosthodontics, Study of Dental Medicine, School of Medicine, University of Split, 21000 Split, Croatia

**Keywords:** sports dentistry, oral health knowledge, athletes, oral hygiene, dental trauma, temporomandibular joint, Croatia

## Abstract

Aim: This study aimed to assess self-reported oral and orofacial health, hygiene habits, and oral health knowledge among Croatian athletes, and to determine factors influencing that knowledge. Differences between contact and non-contact sports, as well as the occurrence of dental trauma and temporomandibular joint (TMJ) symptoms, were also examined. Methods: A cross-sectional, questionnaire-based study was conducted among 1007 athletes (56% male, 44% female) aged 18–42 years, recruited through national sports federations and university sports clubs. The instrument comprised 85 items divided into five domains: sociodemographic data, oral hygiene habits, self-assessed oral health, TMJ symptoms, and oral health knowledge. Data were analysed using descriptive statistics, Chi-square and Fisher’s exact tests, and generalised linear modelling (*p* < 0.05). Results: Athletes demonstrated moderate oral health knowledge (mean score 11.3 ± 4.4/18). While 92.2% recognised that poor oral hygiene leads to caries and periodontitis, only 52.4% correctly identified the ideal time to replant an avulsed tooth. Female participants, older age groups, and those with higher education had significantly better knowledge (*p* ≤ 0.05). Recreational athletes scored higher than amateurs (*p* = 0.002), and those with prior dental trauma experience also showed greater awareness (*p* = 0.028). No significant difference was found between contact and non-contact sports (*p* = 0.287). Despite good brushing habits (86.9% brushed twice daily), only 25.4% regularly used dental floss or interdental brushes. A small proportion of athletes reported symptoms related to temporomandibular joint function, most commonly joint clicking (18.2%), tooth wear (13.4%), and nocturnal bruxism (14.3%). There were no significant differences between contact and non-contact sports, except for muscle stiffness near the temples (*p* = 0.024) and daytime or stress-related teeth grinding (*p* = 0.013 and *p* = 0.018). Conclusions: Croatian athletes demonstrated moderate oral health knowledge and satisfactory hygiene habits, but preventive practices remain inadequate. Education level, gender, and previous dental trauma were key determinants of knowledge. Systematic preventive programmes and targeted education are necessary to improve oral health awareness in sports populations.

## 1. Introduction

Success in sports depends on general health status, training methods, and nutrition—factors that interact to achieve optimal performance. Maintaining adequate oral hygiene is an important component of preserving both oral and overall health in athletes; therefore, routine oral health monitoring should be an integral part of medical supervision in sports [1]. The growing interest in this issue stems from the consistently high prevalence of oral disorders within the athletic population [2].

Sports dentistry, a rapidly developing field within dental medicine, focuses on the prevention, treatment, and management of orofacial conditions [2]. Timely dental care can help maintain oral health, improve dietary habits, fabricate protective mouthguards, and educate athletes on proper oral hygiene, thereby indirectly enhancing athletic performance [3,4]. Dental caries, periodontal diseases, and temporomandibular joint disorders are among the most common pathologies that can adversely affect training capacity and competitive performance. Malocclusion, resulting from genetic predispositions or specific training-related forces, is associated with disturbances in posture, jaw, neck, and back pain, as well as impaired muscle recovery [2,5]. Tooth loss, whether due to trauma or disease, negatively affects digestive function and overall health, as impaired mastication requires greater energy expenditure during digestion. Temporomandibular disorders, such as bruxism, are relatively common in athletic populations and are often linked to psychological stress. Bruxism may cause dental wear and muscle pain in the head, neck, and back, further complicating training and performance [1]. If such conditions remain untreated, they may progress and lead to more severe local or systemic complications, underscoring the importance of regular dental care [2]. Regular oral health monitoring is therefore crucial for maintaining overall health and achieving optimal athletic performance [6]. Frequent consumption of sports and energy drinks, as well as carbohydrate-rich, acidic snacks, increases the risk of dental caries and enamel erosion, partly due to the drop in oral pH and decreased salivary flow that occurs during physical activity [1,4,7,8,9]. International studies indicate a high prevalence of oral diseases among athletes. Research has shown that dental caries, periodontitis, and dental trauma have a significant impact on quality of life and performance during training and competition [10].

Previous scientific research conducted in Croatia has focused on athletes’ knowledge of oral health and the prevalence of dental trauma across different sports, particularly in football, water polo, and handball, as well as in combat sports [11,12,13,14]. These studies generally indicate limited knowledge among athletes regarding dental injuries and oral health [11]. Nevertheless valuable, these studies examined individual sports or specific types of injuries and therefore provide only a partial picture of athletes’ overall oral health status.

Although this study focuses on athletes, challenges related to oral health are not unique to the sporting population. In the general population, oral health knowledge and preventive behaviours are frequently inadequate, and key risk and protective factors associated with caries prevalence—particularly among schoolchildren—illustrate that oral diseases are a widespread public health concern. Such findings indicate persistent gaps in oral health literacy and highlight the importance of education and preventive strategies at all ages. Placing athletes within this broader context reinforces the relevance of examining their oral health knowledge and behaviours [15]. However, broader oral health challenges are not unique to athletes. Studies in the general population show that oral health knowledge and preventive practices are often insufficient, highlighting a wider public health need for improved oral health education.

To date, no study in Croatia has comprehensively assessed oral health knowledge, habits, and self-reported orofacial problems across multiple sports, and has also compared contact and non-contact athletes. Moreover, the relationships among oral health knowledge, injury history, and temporomandibular symptoms remain unclear, as does the extent to which self-reported hygiene habits reflect perceived oral health. Therefore, this study aimed to analyse self-assessed orofacial health, oral hygiene practices, and oral health knowledge among athletes across different sports disciplines, with particular attention to differences between contact and non-contact sports. The study also examined the frequency of temporomandibular joint (TMJ) symptoms and the occurrence of maxillofacial injuries during sports activities. We hypothesised that athletes would demonstrate limited knowledge of oral health and insufficient oral hygiene habits, particularly those participating in contact sports.

## 2. Participants and Methods

### 2.1. Study Design and Participants

This cross-sectional study was conducted at the Department of Endodontics and Restorative Dental Medicine at the University of Split School of Medicine from 1 May to 30 June 2024. The study was approved by the Ethics Committee of the University of Split School of Medicine (Class: 029-01/24-02/0001; No.: 2181-198-03-04-24-0019), 20 February 2024. The research was conducted in accordance with all applicable legal regulations and the principles outlined in the Declaration of Helsinki. The methodology and reporting of the study followed the Checklist for Reporting Results of Internet E-Surveys (CHERRIES) [16].

At the beginning of the questionnaire, all participants were informed of the study’s purpose, the number of questions, the expected duration, the inclusion and exclusion criteria, the research site, and the principal investigators. Participants were also informed that the collected data would be used for a graduate thesis and a scientific publication, that they could withdraw from the study at any time, and that they could contact the investigators with any questions related to the study.

A self-administered online questionnaire (Google Forms) was distributed electronically via social media and e-mail to various sports clubs. Participation in the survey was voluntary and anonymous, and no personal data that could identify respondents was collected. The initial distribution of the questionnaire was followed by one reminder sent four weeks later.

The study included adult athletes of both sexes, with different levels of sports engagement—recreational, amateur, professional athletes, and coaches—who were citizens of the Republic of Croatia and had been actively participating in either contact or non-contact sports for at least three years. Additional inclusion criteria were access to the electronic questionnaire via e-mail or social media, a good command of Croatian, and informed consent to participate in the study. Exclusion criteria included incomplete or inadequately completed questionnaires, as well as failure to meet the inclusion criteria.

According to data from the National Sports Federation (2019), there were 250,964 registered athletes in Croatia [17]. The required sample size was calculated using the Sample Size Calculator (Raosoft, Inc., Seattle, WA, USA) [18]. The minimum sample size (N = 388) was determined based on the total number of registered athletes in Croatia (N = 250,964), with a 95% confidence level, a 5% margin of error, and an assumed response distribution of 50%.

### 2.2. Questionnaire

A self-structured questionnaire comprising 85 items was developed specifically for this study by a specialist in endodontics and restorative dental medicine, in collaboration with a sixth-year dental student. The questionnaire was designed based on relevant scientific literature from related research fields [1,6,10,11,12,14]. The questionnaire aimed to assess oral health status, oral hygiene habits, knowledge of oral health, the presence of temporomandibular joint (TMJ) symptoms, and the frequency of sports-related orofacial injuries. Before the main study, the questionnaire was pilot-tested to evaluate its readability, clarity, and comprehensibility. The pilot study included 20 athletes, and the results indicated that no further modifications were necessary. The average completion time was approximately 15 min. Participants in the pilot study were not included in the main research sample.

The first section of the questionnaire contained seven questions (Q1–Q7) related to sociodemographic characteristics, including gender, age, education level, family socioeconomic status, height, and body weight, which were used to calculate the Body Mass Index (BMI). The second section comprised 18 items (Q8–Q25) that assessed athletes’ knowledge of oral health. Each question offered three possible answers: “Yes,” “No,” and “I do not know.” A “Yes” answer was considered correct, while “No” and “I do not know” were classified as incorrect. One point was awarded for each correct answer, with a maximum possible score of 18. According to Bloom’s taxonomy, overall knowledge was categorised as good (80–100%, 14.4–18.0 points), moderate (60–79%, 10.8–14.3 points), or poor (<60%, <10.8 points) [19]. The third section (Q26–Q32) addressed sports-related characteristics, including weekly training hours, level of participation (recreational or competitive), and type of sport (contact or non-contact). It also included questions about the type and location of sports-related injuries. The fourth section consisted of 21 questions (Q33–Q53) assessing self-reported TMJ symptoms among athletes. Respondents answered “Yes,” “No,” or “I do not know”; “Yes” was classified as a positive response, while “No” and “I do not know” were considered negative responses. The fifth section contained eight questions (Q54–Q61) related to oral hygiene habits, including the frequency and methods of oral hygiene practices and dental visits. The sixth and final section (Q62–Q85) comprised 24 questions focused on oral health problems and prior dental treatments. These questions included the presence of caries, gingivitis, xerostomia, and burning sensations in the oral mucosa, as well as information about prosthodontic and orthodontic treatments, such as crowns, bridges, implants, and orthodontic therapy.

### 2.3. Statistical Analysis

Data analysis was performed using IBM SPSS Statistics, version 26.0 (IBM Corp., Armonk, NY, USA), with a significance level set at α < 0.05. The normality of variable distribution was assessed using the Kolmogorov–Smirnov test. For descriptive analysis, categorical variables were presented as absolute and relative frequencies (number and percentage). In contrast, numerical variables were expressed as either the median and interquartile range or the mean and standard deviation, depending on the data distribution. Differences between categorical variables for the two analysed groups (contact and non-contact sports) were examined using the Chi-square test or Fisher’s exact test, as appropriate. To identify factors associated with knowledge level, a generalised linear model was applied. Independent variables included gender, age, level of education, socioeconomic status, type of sports participation, contact vs. non-contact sport classification, and history of dental trauma.

## 3. Results

### 3.1. Participant Characteristics

Demographic and professional characteristics of the participants are presented in Table 1. A total of 1007 individuals participated in the study, comprising 56% males and 44% females. Most participants were engaged in contact sports (80.8%), while the remainder participated in non-contact sports (19.2%). Statistically significant differences between contact and non-contact athletes were observed for age, gender, level of education, and body weight (*p* ≤ 0.05 for all comparisons).

### 3.2. Oral Health Knowledge

Participants’ oral health knowledge is summarised in Table 2. The highest proportion of correct responses was observed for statements regarding the relationship between poor oral hygiene and dental diseases, as well as the impact of oral health on quality of life. Contact and non-contact athletes differed significantly only in their knowledge regarding the immediate management of tooth avulsion (*p* ≤ 0.001).

### 3.3. Sports Participation and Injury Profile

Table 3 presents characteristics of sports participation, as well as the frequency and types of injuries. Among the sample, 12.3% were coaches, 15.7% were professional athletes, 41.2% were amateur athletes, and 30.8% were recreational athletes. Contact athletes reported higher rates of musculoskeletal injuries, fractures/dislocations, concussions, facial, jaw, and dental trauma compared to non-contact athletes (all *p* ≤ 0.05).

### 3.4. Temporomandibular Joint Symptoms

TMJ-related symptoms and parafunctional habits are summarised in Table 4. The most frequently reported symptom was a “ringing” sensation in the ear (19.9%), followed by nail-biting (25.1%) and nocturnal teeth grinding (14.4%). Significant differences between contact and non-contact athletes were observed for muscle stiffness in the temporal region and certain parafunctional habits, including daytime and stress-related teeth grinding (*p* ≤ 0.05).

### 3.5. Oral Hygiene Habits

Participants’ oral hygiene practices are presented in Table 5. Most respondents brushed their teeth twice daily (86.9%) and for at least two minutes (77.9%). More than half cleaned their tongue while brushing (53.1%) and attended regular dental check-ups (50.9%). Contact and non-contact athletes differed significantly only in the use of dental floss or interdental brushes (*p* = 0.035).

### 3.6. Self-Reported Oral Health Problems

Data on oral health problems and treatments over the past six months are shown in Table 6. The most commonly reported issues were gum bleeding during brushing (42.7%), dental calculus (37.9%), and dry mouth (33.7%). Significant differences between contact and non-contact athletes were observed for dental trauma, calculus, tooth discoloration, gingival recession, and orthodontic treatment (*p* ≤ 0.05 for all).

### 3.7. Overall Oral Health Knowledge

Table 7 presents overall oral health knowledge scores according to demographic and professional characteristics. Female participants and those with higher levels of education demonstrated greater knowledge (*p* ≤ 0.05). Recreational athletes had the highest knowledge scores, which were significantly higher than those of amateur athletes (*p* = 0.002). Participants with prior facial, jaw, or dental trauma also had higher knowledge scores (*p* = 0.028). No significant differences in overall knowledge were observed between contact and non-contact athletes. Mean knowledge scores ranged from 10.6 ± 4.5 in amateurs to 11.9 ± 4.2 in recreational athletes, with 3% of participants not answering any questions correctly and 9.8% answering all questions correctly.

## 4. Discussion

### 4.1. Knowledge and Education

This study aimed to assess self-perceived orofacial health, oral hygiene habits, and oral health knowledge among athletes with varying levels of sports involvement. Overall, athletes demonstrated a moderate level of oral health knowledge, thereby rejecting the initial hypothesis that knowledge would be limited. However, despite a satisfactory theoretical understanding, translating knowledge into daily practice was insufficient, as more than one-quarter of participants reported gum bleeding, unpleasant breath, or dry mouth. These findings highlight the need for additional education not only about oral health, but also about its broader relevance to athletic performance and general well-being. Similar results have been observed in studies among Croatian water polo players and football players, who also showed limited knowledge of key oral health concepts, particularly those related to emergency management of traumatic dental injuries [11,12].

The present study found no significant differences in overall oral health knowledge between athletes in contact and non-contact sports. Among professional categories, recreational athletes had the highest knowledge scores, significantly higher than amateur athletes. This finding may be explained by differences in age distribution, health awareness, or educational exposure: recreational athletes in this sample tended to be older and potentially more health-conscious, whereas amateur athletes often represent a younger group with less experience and less exposure to formal health education—the observed associations between higher educational attainment and better knowledge support this interpretation. Similarly, female participants demonstrated higher knowledge scores than male participants, which aligns with the literature indicating that women more frequently use dental services and engage in preventive health behaviours. It is also important to acknowledge that some potentially relevant sociodemographic factors—such as income level, access to dental care, and urban versus rural residence—were not assessed in this study. However, they are known to influence oral health knowledge [15]. Consistent with earlier studies, sociodemographic factors, such as educational level, were strongly associated with knowledge: participants with higher education attained higher scores, mirroring findings from Croatian studies on football and water polo players [11,12]. In this study, athletes aged 25–31 years demonstrated the highest levels of knowledge, whereas previous research found that younger athletes were better informed [12]. Despite moderate overall knowledge, misconceptions persisted about the management of avulsed teeth: fewer than one-third knew that replantation is possible, and only one-quarter knew the correct handling and cleaning procedures. Such gaps reinforce the need for targeted educational interventions.

### 4.2. Oral Hygiene Behaviours and Oral Health Issues

Although most athletes reported brushing their teeth at least twice per day (86.9%) and for more than two minutes (77.9%), symptoms indicative of inadequate oral hygiene practices remained common. Gingival bleeding was reported by 42.7% of respondents, while more than one-third experienced dental calculus or dry mouth. These findings align with international data indicating that self-reported brushing frequency does not necessarily translate into satisfactory oral health outcomes. For example, a study among professional British athletes found high rates of untreated caries (49%) and gingivitis (77%) despite 94% brushing twice daily [20]. Similarly, athletes at the Pan American Games in Lima showed high prevalence of untreated caries (29%) and periodontal inflammation (34%) [2].

Insufficient use of interdental cleaning tools and mouthwashes may partially explain the persistent prevalence of gingival inflammation: fewer than one-quarter of athletes reported using interdental brushes or dental floss (25.4%), and only 20.7% used antiseptic mouthwash. This “knowledge–behaviour gap” has been confirmed across various sports populations and underscores the importance of integrating oral health promotion into sports medicine systems, which remains limited in practice [20].

Dry mouth was reported by 33.7% of participants—likely related to dehydration, intense training, and stress—and may further predispose athletes to caries, enamel erosion, and halitosis. These symptoms are not merely aesthetic concerns; oral diseases may directly impair performance. Between 5% and 18% of athletes in previous studies reported that oral pain, discomfort, and related psychological stress negatively affected their training and competition outcomes [21].

### 4.3. Injuries, Trauma, and Preventive Practices

Athletes are inherently exposed to a higher risk of dental trauma due to physical contact, rapid movements, and increased collision frequency [11,12]. In this study, 40% reported at least one dental or orofacial injury during their sporting career. The most common injuries were tooth damage, soft tissue injuries, and tongue trauma. Comparable prevalence has been reported among skiers [22], football players [11], water polo players [12], and professional handball players in Switzerland, where 40.8% had experienced dental trauma—most commonly crown fractures [23].

Despite the well-documented protective role of sports mouthguards, their use remains low. Previous studies indicate an apparent discrepancy between awareness and practice: although 67% of handball players in Croatia believed mouthguards reduce injury risk, only 28% used them regularly [24]. The present findings echo this pattern, reinforcing the need for stronger preventive education and more consistent implementation of protective strategies across sports.

### 4.4. Temporomandibular Joint (TMJ) Symptoms and Stress-Related Findings

Temporomandibular disorders (TMD) represent an emerging concern in athletic populations, particularly among those exposed to high physical strain or frequent contact [25]. In this study, 14.3% reported nighttime tooth grinding and 10.5% reported stress-induced bruxism, both of which can exacerbate TMJ dysfunction. Physical exertion, especially in sports that require intense weightlifting, often leads to involuntary tooth clenching, further increasing stress on the TMJ [25]. Studies among weightlifters similarly report high prevalence of bruxism and sensations of occlusal discomfort [25].

In the present study, the most frequently reported TMJ-related symptoms were crepitation on mouth opening (18.2%) and ear pain or “ringing” (20%). These findings are comparable to other athletic populations, although some studies have not found significant differences in TMD prevalence between athletes and non-athletes [26]. Nevertheless, emerging evidence suggests that protective dental splints may alleviate TMJ symptoms in athletes exposed to repeated head impacts, indicating potential value for targeted preventive interventions [27].

### 4.5. Practical Implications and Study Limitations

A significant strength of this study is its large sample size—over 1000 athletes and coaches—providing robust insight into oral health across various sports. Nonetheless, several limitations must be acknowledged. The use of a self-administered questionnaire introduces potential for socially desirable responding and inaccuracies due to misunderstanding of specific terms. Questionnaire length may have contributed to participant fatigue, potentially affecting response accuracy in later sections. Selection bias cannot be excluded, as individuals more interested in the topic may have been more likely to participate. Additionally, the cross-sectional design limits causal interpretation and prevents assessment of changes in oral health or behaviour over time.

Future research should incorporate longitudinal designs and clinical examinations to complement self-reported data, enabling more objective evaluation of oral health status in athletic populations. Integrating dental professionals into sports medicine teams and implementing structured educational programs could improve oral health outcomes and athletic performance.

## 5. Conclusions

The findings of this study indicate that Croatian athletes and coaches generally have moderate oral health knowledge, with good awareness of the impact of poor oral hygiene but limited understanding of the emergency management of dental injuries. Although most participants reported regular brushing and dental visits, the low use of interdental cleaning tools and frequent symptoms such as bleeding gums, dental calculus, and dry mouth highlight a clear gap between knowledge and effective daily practice. Contact-sport athletes reported more dental trauma, yet their knowledge levels did not differ significantly from those in non-contact sports. Higher education and female gender were associated with better knowledge.

These results have important clinical and practical implications. The discrepancy between knowledge and behaviour suggests a need for improved preventive strategies within sports environments. Incorporating structured oral health education, emphasising practical hygiene techniques and dental trauma management, and integrating routine dental screenings within sports medicine could substantially improve athletes’ oral health. Strengthening these preventive measures may help reduce the burden of oral disease and support better performance and overall well-being among athletes.

## Figures and Tables

**Table 1:** Demographic and Professional Characteristics of the Participants.

Characteristics		Total N = 1007	Sport Contact Type		*p*-Value
			Contact Sports N = 841	Non-Contact Sports N = 166	
Age	18–24	626 (62.2)	540 (66.2)	86 (51.9)	0.001
	25–31	231 (22.9)	188 (23.0)	43 (25.9)	
	>31	150 (14.9)	113 (13.8)	37 (22.2)	
Sex	Male	561 (55.8)	486 (58.8)	75 (45.2)	0.003
	Female	446 (44.2)	355 (41.2)	91 (54.8)	
Level of Education	Secondary School	421 (41.8)	369 (45.3)	52 (31.3)	0.001
	College	300 (29.8)	256 (31.4)	44 (26.5)	
	University	235 (23.3)	181 (22.2)	54 (32.5)	
	MSc/PhD	51 (5.1)	35 (4.3)	16 (9.6)	
Family Socioeconomic Status	Below Average	43 (4.3)	40 (4.9)	3 (1.8)	0.146
	Average	793 (78.7)	693 (85.1)	130 (78.3)	
	Above Average	171 (16.9)	138 (16.9)	33 (19.8)	
Height of Participants (cm)	<170	216 (21.4)	182 (22.3)	34 (20.4)	0.508
	171–190	644 (63.9)	532(65.3)	112 (67.4)	
	>190	147 (14.6)	127 (15.6)	20 (12.0)	
Weight of Participants (kg)	<70	334 (33.2)	265 (32.6)	69 (41.6)	0.022
	71–90	457 (45.4)	386 (47.4)	71 (42.7)	
	>90	216 (21.4)	190 (23.3)	26 (15.7)	
BMI	<18	8 (0.8)	8 (0.8)	0 (0.0)	0.056
	18–25	615 (61.0)	501 (49.7)	114 (68.7)	
	>25	384 (38.1)	332 (40.7)	52 (31.3)	

Values are presented as number and percentage. *p*-values were analysed using the Chi-square test or Fisher’s exact test at α = 0.05.

**Table 2:** Distribution of Correct Answers to Questions Assessing Participants’ Knowledge of Oral Health.

Characteristic	Total N = 1007	Sport Contact Type		*p*-Value
		Contact Sports N = 841	Non-Contact Sports N = 166	
Oral health is closely related to an individual’s general health.	848 (84.2)	703 (83.6)	145(87.3)	0.251
Oral health is closely related to an individual’s quality of life.	857 (85.1)	710 (84,4)	147 (88.6)	0.172
The most common oral diseases are dental caries, periodontitis, and oral cancer.	714 (70.9)	597 (71.0)	117 (70.5)	0.896
Poor oral hygiene can lead to the development of dental caries and periodontitis.	928 (92.2)	772 (91.8)	156 (94.0)	0.343
Diet has an influence on the development of dental caries, periodontitis, and oral cancer.	826 (82.0)	688 (81.8)	138 (83.1)	0.684
Smoking is associated with oral cancer and periodontal diseases.	854 (84.8)	708 (84.2)	146 (88.0)	0.217
A high alcohol intake is associated with an increased risk of developing oral cancer, periodontitis, and dental caries.	630 (62.6)	522 (62.1)	108 (65.1)	0.467
Sports drinks and energy beverages can damage the tooth surface and cause dental erosion.	531 (52.7)	440 (52.3)	91 (54.8)	0.555
Mouthguards are an effective means of preventing dental injuries during sports activities.	845 (83.9)	703 (83.6)	142 (85.5)	0.556
An avulsed tooth is one that has been completely displaced from its alveolar socket and is entirely out of the oral cavity.	465 (46.2)	387 (46.0)	78 (47.0)	0.797
Permanent teeth that have been avulsed due to trauma can be replanted (reinserted) into the oral cavity.	348 (34.6)	299 (35.6)	49 (29.5)	0.135
The emergency treatment for a tooth that has been completely avulsed from its alveolar socket and contaminated with debris involves rinsing the tooth under running water and replanting it into its socket in the oral cavity.	260 (25.8)	225 (26.8)	35 (21.1)	0.127
An avulsed tooth should be handled by the crown during replantation and manipulation.	250 (24.8)	211 (25.1)	39 (23.5)	0.664
If it is not possible to replant the tooth at the site of the accident, it should be stored in a moist medium (milk or specialised tooth preservation solutions) until reaching a dentist.	335 (33.3)	276 (32.8)	59 (35.5)	0.496
The ideal time to seek professional care in the event of tooth avulsion is immediately, within 30 min of the injury.	528 (52.4)	426 (50.7)	102 (61.4)	≤0.001
The upper anterior teeth are most frequently affected by injuries.	689 (68.4)	574 (68.3)	115 (69.3)	0.795
Teeth should be brushed twice daily for at least two minutes using fluoride toothpaste.	846 (84.0)	701 (83.4)	145 (87.3)	0.199
Fluorides play a protective role against caries by preventing damage to the tooth surface, aiding in remineralization, and inhibiting bacterial growth.	669 (66.4)	549 (65.3)	120 (72.3)	0.083

Values are presented as number and percentage. *p*-values were analysed using the Chi-square test or Fisher’s exact test at α = 0.05.

**Table 3:** Characteristics of Sports Participation and Injuries Sustained during Sports Activities.

Characteristic		Total N = 1007	Sport Contact Type		*p*-Value
			Contact Sports N = 841	Non-Contact Sports N = 166	
Number of training hours per week	0–5	591 (58.6)	476 (58.4)	115 (69.2)	0.011
	6–10	325 (32.3)	285 (33.7)	40 (24.1)	
	>10	91 (9.1)	80 (7.9)	11 (6.7)	
Type of sports participation	Recreational	310 (30.8)	282 (27.1)	82 (49.4)	0.032
	Amateur	415 (41.2)	367 (43.6)	48 (28.9)	
	Professional	158 (15.7)	148 (17.6)	10 (6.0)	
	Coaching	124 (12.3)	98 (11.7)	26 (15.7)	
Injury to bones, muscles, ligaments, or tendons		807 (80.1)	685 (84.1)	122 (73.4)	0.026
Fracture/dislocation		559 (55.5)	497 (61.0)	62 (37.3)	<0.001
Concussion		123 (12.2)	119 (14.6)	4 (2.4)	<0.001
Facial, jaw, or dental trauma during sports activities		305 (30.3)	282 (34.6)	23 (13.8)	0.001
Dental injury		139 (17.1)	125 (15.3)	14 (8.4)	0.038

Values are presented as number and percentage. *p*-values were analysed using the Chi-square test or Fisher’s exact test at α = 0.05.

**Table 4:** Temporomandibular Joint Symptoms.

Symptom		Total N = 1007	Sport Contact Type		*p*-Value
			Contact Sports N = 841	Non-Contact Sports N = 166	
Disorders of the jaws or temporomandibular joint	Yes	50 (4.9)	38 (4.5)	12 (7.2)	0.142
Audible or palpable clicking or crepitation in the temporomandibular joints during mouth movements	Yes	183 (18.2)	154 (18.3)	29 (17.5)	0.797
Limitations or discomfort associated with chewing	Yes	43 (4.3)	37 (4.4)	6 (3.6)	0.648
Asymmetric mouth opening	Yes	68 (6.8)	60 (7.1)	8 (4.8)	0.277
Difficulty in maximal mouth opening	Yes	49 (4.9)	41 (4.9)	8 (4.8)	0.976
Muscle stiffness and/or pain in the muscles around the temples	Yes	28 (2.8)	19 (2.3)	9 (5.4)	0.024
Discomfort and/or limitations associated with mouth opening	Yes	39 (3.9)	30 (3.6)	9 (5.4)	0.258
Discomfort and/or limitations associated with mouth closing	Yes	13 (1.3)	9 (1.1)	4 (2.4)	0.247
Discomfort and/or limitations with lateral jaw movements	Yes	45 (4.5)	37 (4.4)	8 (4.8)	0.811
Muscle stiffness and/or pain in the muscles around the mandibular angle	Yes	31 (3.1)	24 (2.9)	7 (4.2)	0.353
Worn upper and/or lower teeth	Yes	135 (13.4)	108 (12.8)	27 (16.3)	0.237
Nocturnal teeth grinding	Yes	144 (14.3)	115 (13.7)	29 (17.5)	0.202
Daytime teeth grinding	Yes	43 (4.3)	30 (3.6)	13 (7.8)	0.013
Stress-related teeth grinding	Yes	106 (10.5)	80 (9.5)	26 (15.7)	0.018
Jaw fracture	Yes	13 (1.3)	12 (1.4)	1 (0.6)	0.493
Frequent headaches or migraines (more than once a week, of unknown origin)	Yes	105 (10.4)	90 (10.7)	15 (9)	0.521
Ear pain or “ringing” sensation or a feeling of fullness in the ear	Yes	201 (19.9)	163 (19.4)	38 (22.9)	0.301
Pain in front of the ear radiating to the cheek, ear, and temple	Yes	25 (2.5)	19 (2.3)	6 (3.6)	0.305
Missing posterior teeth	Yes	120 (11.9)	102 (12.1)	18 (10.8)	0.641
Parafunctional habits such as nail biting and holding objects between the teeth	Yes	253 (25.1)	220 (26.2)	33 (19.9)	0.088
Frequent chewing of chewing gum	Yes	333 (33.1)	286 (35.1)	47 (28.3)	0.154

Values are presented as number and percentage. *p*-values were analysed using the Chi-square test or Fisher’s exact test at α = 0.05.

**Table 5:** Oral Hygiene Habits for Maintaining Oral Health.

Habits		Total N = 1007	Sport Contact Type		*p*-Value
			Contact Sports N = 841	Non-Contact Sports N = 166	
Brushing teeth two or more times a day	Yes	875 (86.9)	736 (87.5)	139 (83.7)	0.187
Brushing duration of two or more minutes	Yes	784 (77.9)	658 (78.2)	126 (75.9)	0.508
Use of fluoride toothpaste	Yes	696 (69.10)	574 (68.3)	122 (73.5)	0.182
Tongue cleaning while brushing teeth	Yes	535 (53.1)	453 (53.9)	82 (49.4)	0.292
Use of mouthwash as part of daily oral hygiene	Yes	208 (20.70)	169 (20.1)	39 (23.5)	0.323
Daily use of dental floss or interdental brush	Yes	256 (25.4)	203 (24.1)	53 (31.9)	0.035
Regular dental check-ups every six months	Yes	513 (50.9)	429 (50.1)	84 (50.6)	0.923
Replacing the toothbrush every 2 to 3 months	Yes	833 (82.7)	700 (83.2)	133 (80.1)	0.332

Values are presented as number and percentage. *p*-values were analysed using the Chi-square test or Fisher’s exact test at α = 0.05.

**Table 6:** Dental or Oral Cavity Problems and Treatments in the Past Six Months.

Oral Cavity Problems and Treatments		Total N = 1007	Sport Contact Type		*p*-Value
			Contact Sports N = 841	Non-Contact Sports N = 166	
Toothache	Yes	251 (24.9)	210 (25.0)	41 (24.7)	0.941
Gum bleeding during brushing	Yes	430 (42.7)	361 (42.9)	69 (41.6)	0.746
Swollen and painful gums	Yes	212 (21.1)	172 (20.5)	40 (24.1)	0.293
Bad breath	Yes	277 (27.5)	228 (27.1)	49 (29.5)	0.526
Burning and stinging sensation of the oral mucosa	Yes	31 (3.1)	25 (3.0)	6 (3.6)	0.662
Dry mouth	Yes	339 (33.7)	285 (33.9)	54 (32.5)	0.735
Swelling	Yes	49 (4.9)	42 (5.0)	7 (4.2)	0.671
Loose tooth	Yes	35 (3.5)	28 (3.3)	7 (4.2)	0.568
Difficulty chewing and eating	Yes	91 (9.0)	77 (9.2)	14 (8.4)	0.767
Fractured or avulsed tooth	Yes	60 (6.0)	44 (5.2)	16 (9.6)	0.028
Blisters or aphthae on the oral mucosa	Yes	212 (21.1)	174 (20.7)	38 (22.9)	0.525
Difficulty in speaking	Yes	27 (2.7)	25 (3.0)	2 (1.2)	0.292
Pain in the temporomandibular joint (TMJ)	Yes	36 (3.6)	28 (3.3)	8 (4.8)	0.345
Dental caries	Yes	287 (28.5)	235 (27.9)	52 (31.3)	0.378
Dental calculus	Yes	382 (37.9)	306 (36.4)	76 (45.8)	0.023
Tooth discoloration	Yes	158 (15.7)	123 (14.6)	35 (21.1)	0.037
Gingival recession at the tooth neck	Yes	108 (10.7)	76 (9.0)	32 (19.3)	0.001
Dental filling	Yes	268 (26.6)	220 (26.2)	48 (28.9)	0.463
Endodontic treatment	Yes	61 (6.1)	50 (5.9)	11 (6.6)	0.737
Tooth extraction	Yes	60 (6.0)	49 (5.8)	11 (6.6)	0.691
Orthodontic treatment	Yes	91 (9.0)	84 (10.0)	7 (4.2)	0.018
Prosthetic treatment	Yes	31 (3.1)	24 (2.9)	7 (4.2)	0.353
Periodontal treatment	Yes	21 (2.1)	19 (2.3)	2 (1.2)	0.556
Dental implant	Yes	22 (2.2)	20 (2.4)	2 (1.2)	0.409

Values are presented as number and percentage. *p*-values were analysed using the Chi-square test or Fisher’s exact test at α = 0.05.

**Table 7:** Demographic and Professional Characteristics of Participants in Relation to Overall Oral Health Knowledge.

Characteristic	Answer	Oral Health Knowledge *β* (95% CI)	*p*-Value
Sex	Male	Reference	
	Female	1.034 (0.412–1.657)	0.001
Age group	18–24	Reference	
	25–31	0.890 (0.221–1.559)	0.009
	>31	0.571 (−0.138–1.277)	0113
Level of education	Secondary School	Reference	
	College	0.652 (0.009–1.295)	0.047
	University	0.850 (0.071–1.629)	0.032
	MSc/PhD	1.523 (0.149–2.896)	0.030
Socioeconomic Status	Below average	Reference	
	Average	0.170 (−1.151–1.490)	0.801
	Above average	0.520 (−0.932–1.973)	0.482
Type of Sport by Contact Level	Non-contact	Reference	
	Contact	0.402 (−1.149–0.340)	0.287
Type of Sports Participation	Recreational	Reference	
	Amateur	−1.076 (−1.752–0.400)	0.002
	Professional	−0.435 (−1.313–0.443)	0.332
	Coaching	−0.302 (−1300–0.697)	0.554
Experience of Dental Trauma	No	Reference	
	Yes	0.672 (0.074–0.271)	0.028

Data are presented as β (95% CI). Abbreviations: β—regression coefficient, 95% CI—95% confidence interval.

## Data Availability

Due to ethical considerations, we are unable to share the original data. However, the data presented in this study are available on request from the corresponding author.
